# A systems-level analysis of the mutually antagonistic roles of RKIP and BACH1 in dynamics of cancer cell plasticity

**DOI:** 10.1098/rsif.2023.0389

**Published:** 2023-11-15

**Authors:** Sai Shyam, Soundharya Ramu, Manas Sehgal, Mohit Kumar Jolly

**Affiliations:** Department of Bioengineering, Indian Institute of Science, Bangalore 560012, India

**Keywords:** phenotypic plasticity, systems biology, metabolic reprogramming, cancer metastasis

## Abstract

Epithelial–mesenchymal transition (EMT) is an important axis of phenotypic plasticity—a hallmark of cancer metastasis. Raf kinase-B inhibitor protein (RKIP) and BTB and CNC homology 1 (BACH1) are reported to influence EMT. In breast cancer, they act antagonistically, but the exact nature of their roles in mediating EMT and associated other axes of plasticity remains unclear. Here, analysing transcriptomic data, we reveal their antagonistic trends in a pan-cancer manner in terms of association with EMT, metabolic reprogramming and immune evasion via PD-L1. Next, we developed and simulated a mechanism-based gene regulatory network that captures how RKIP and BACH1 engage in feedback loops with drivers of EMT and stemness. We found that RKIP and BACH1 belong to two antagonistic ‘teams’ of players—while BACH1 belonged to the one driving pro-EMT, stem-like and therapy-resistant cell states, RKIP belonged to the one enabling pro-epithelial, less stem-like and therapy-sensitive phenotypes. Finally, we observed that low RKIP levels and upregulated BACH1 levels associated with worse clinical outcomes in many cancer types. Together, our systems-level analysis indicates that the emergent dynamics of underlying regulatory network enable the antagonistic patterns of RKIP and BACH1 with various axes of cancer cell plasticity, and with patient survival data.

## 1. Introduction

Cancer metastasis is driven by phenotypic plasticity—the dynamic and reversible adaptation of disseminating cancer cells to different microenvironments that they encounter along the journey. Phenotypic switching among the epithelial (E), mesenchymal (M) and hybrid E/M phenotypes through epithelial to mesenchymal transition (EMT) and its reverse mesenchymal to epithelial transition (MET) constitute an important axis of phenotypic plasticity during metastasis [1,2]. Metabolic reprogramming is another key axis of plasticity and a hallmark of cancer metastasis [3]. Different axes of plasticity—EMT/MET, metabolic switching, and immune evasion—are often interconnected, thus enabling cancer cells to exist in distinct cell states [4–6], and promoting phenotypic plasticity and consequent non-genetic heterogeneity.

Understanding the dynamics of the interconnected axes of plasticity is critical to restrict metastasis. For instance, EMT often leads to increased levels of PD-L1—a transmembrane molecule leading to the immune escape of cancer cells [6–8]. Further, the knockdown of PD-L1 could reverse EMT [9], and its upregulation can promote EMT [10,11]. Similarly, EMT and tamoxifen resistance in ER+ breast cancer cells can drive each other [12]. Such bidirectional connections are often mediated by multiple feedback loops among the molecules driving cell plasticity along these multiple axes. Breaking the miR-200/ZEB1 mutually inhibitory feedback loop in breast cancer cells through CRISPR/Cas9 can reduce cancer cell metastasis [13]. Thus, mapping the different feedback loops that can govern cell plasticity is of fundamental importance.

Recent reports have identified a mutually inhibitory feedback loop between the Raf kinase inhibitor protein (RKIP) and BTB and CNC homology 1 (BACH1) in the regulation of EMT and metastasis in breast cancer [14,15]. BACH1 represses RKIP transcriptionally, and RKIP can inhibit BACH1 via microRNA let-7. RKIP is a metastasis suppressor that acts along the RAF1/MEK/ERK pathway to regulate cell proliferation and migration in prostate cancer, breast cancer, lung cancer and pancreatic cancer [16–18]. Its exogenous expression in metastatic breast cancer cells can suppress invasion, intravasation and metastasis in xenograft mouse models [19,20]. On the other hand, BACH1 can promote metastasis in breast cancer, ovarian cancer, lung cancer and pancreatic cancer [21–25]. While their mutual antagonism in breast cancer has been reported, it remains unclear whether this antagonism is seen in other cancers and how these molecules regulate different axes of cellular plasticity implicated in metastasis.

Here, we first investigated whether RKIP and BACH1 show antagonistic trends across different cancer types using transcriptomic data from The Cancer Genome Atlas (TCGA). We found RKIP and BACH1 to be anti-correlated with each other in most cancer types. Moreover, while BACH1 correlated positively with EMT and PD-L1 but negatively with oxidative phosphorylation and fatty acid oxidation, RKIP showed opposite trends in a pan-cancer manner. These trends were recapitulated in ER+ breast cancer datasets, where BACH1 also correlated negatively with ESR1 (estrogen receptor), but RKIP correlated positively with it. To better understand these consistent patterns in transcriptomic signatures, we constructed and simulated a mechanism-based gene regulatory network (GRN) that incorporated the feedback loops formed among RKIP, BACH1 and other master regulators of cancer cell plasticity such as ZEB1, miR-200, LIN28 and let-7. Our analysis of GRN identified that RKIP and BACH1 belonged to two mutually repressing ‘teams’ of players—one that was comprised of pro-EMT (ZEB1, LIN28, SNAIL, SLUG) players, and the other constituted pro-MET (miR-200, let-7, miR-145, CDH1) ones. These ‘teams’ enable the BACH1-high cells to display a hybrid E/M and/or mesenchymal state exhibiting a stem-like behaviour, as well as opposite trends in terms of association of RKIP and BACH1 with patient survival across cancer types. Together, our results explain the emergent dynamics of underlying GRN that can underlie the observed antagonistic behaviour of RKIP and BACH1 in a pan-cancer manner.

## 2. Results

### 2.1. RKIP and BACH1 display mutual antagonistic patterns across many cancer types in TCGA

**Figure 1.**
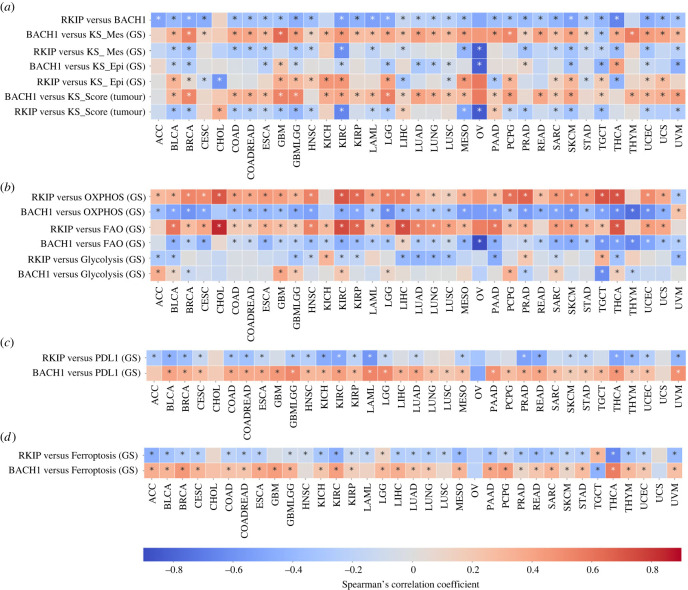
Correlation of RKIP and BACH1 expression with EMT, metabolic reprogramming and immune evasion signatures in TCGA. Heatmaps representing Spearman's correlation coefficient of RKIP and BACH1 with (*a*) each other, KS Score and ssGSEA scores of KS-Epi and KS-Mes gene signatures, (*b*) ssGSEA scores of hallmark gene signatures (GS) for OXPHOS, glycolysis and FAO, (*c*) ssGSEA scores for PDL1 gene signature (GS) and (*d*) ssGSEA scores for ferroptosis gene signature (GS). *p-value < 0.05.

RKIP and BACH1 have been reported as mutually inhibitory players in breast cancer; while RKIP is anti-metastatic, BACH1 is pro-metastatic [14]. To investigate whether they show antagonistic trends consistently across other cancers, we analysed TCGA data from 35 different cancer types. RKIP and BACH1 were found to be negatively correlated with each other in 31 out of 35 (88.57%) cancer types (figure 1*a*). To understand their association with EMT, we calculated the ssGSEA score for each sample for the KS-Epithelial (KS-Epi) and KS-Mesenchymal (KS-Mes) signatures. KS-Epi signature comprises genes that are upregulated in epithelial cells in a pan-cancer manner, while genes in KS-Mes signatures are upregulated in mesenchymal cells [26]. The ssGSEA score of KS-Epi signature correlated positively with RKIP levels in only 15 cancer types and negatively with BACH1 expression in 14 cancer types, thus indicating a rather ambivalent association. On the other hand, the KS-Mes scores were negatively correlated with RKIP levels in 57.14% (20 out of 35) cancer types but positively correlated with BACH1 expression in 88.57% (31 out of 35) of cases (figure 1*a*). Further, RKIP showed a negative correlation with KS EMT score in 57.14% (20 out of 35) of cancers but BACH1 showed a positive correlation with it in 77.14% (27 out of 35) of cases. The higher the KS EMT score, the more mesenchymal the sample is [27]. Together, these results suggest that RKIP and BACH1 show antagonistic trends in a pan-cancer manner; RKIP associates with an epithelial phenotype while BACH1 with a mesenchymal phenotype.

During EMT, cells can also exhibit metabolic plasticity, typically leading to decreased oxidative phosphorylation and fatty acid oxidation but increased glycolysis [28]. To investigate how RKIP and BACH1 expression levels correlate with these pathways, we calculated the ssGSEA scores for OXPHOS, FAO and glycolysis gene signatures in TCGA samples. OXPHOS ssGSEA scores correlated positively with RKIP in 88.57% (31 out of 35) of the cancer types, while BACH1 correlated negatively in 91.14% (32 out of 35) of the cancer types (figure 1*b*). Similar trends were observed with FAO, with RKIP correlating positively and BACH1 correlating negatively with the corresponding ssGSEA scores in 31 out of 35 cancer types. On the other hand, ssGSEA scores for glycolysis signature did not show such strong trends—they correlated negatively with RKIP in 14 cancer types, and positively with BACH1 in 9 of them. Also, while FAO and OXPHOS ssGSEA scores correlated positively with each other, glycolysis did not show consistent negative correlations with either of them (electronic supplementary material, figure S1*a*). Overall, RKIP and BACH1 exhibited opposite patterns in terms of their association with OXPHOS and FAO.

Another axis of phenotypic plasticity coupled with EMT is immune evasion. As cells undergo a partial or complete EMT, they display increased levels of PD-L1, an immune checkpoint molecule that evades attack by the immune system [4,29]. Thus, we probed how the expression levels of PD-L1 and scores for PD-L1 gene signature correlate with RKIP and BACH1. We observed that among 35 cancer types, PD-L1 expression correlated negatively with RKIP expression in 22 of them and positively with BACH1 in 32 of them (electronic supplementary material, figure S1*b*). Similarly, the ssGSEA scores of PD-L1 gene signature correlated negatively with RKIP in 68.57% (24 out of 35) but positively with BACH1 in 88.57% (31 out of 35) of the cancer types (figure 1*c*). Further, cells undergoing EMT have been shown to be vulnerable to ferroptosis, an iron-dependent cell death programme [30]. To examine how RKIP and BACH1 associated with ferroptosis, we calculate the ssGSEA scores of a ferroptosis-based gene signature in cancer [31]. Consistently, we found that RKIP associates negatively with it, but BACH1 associates positively (figure 1*d*). Similar trends were observed when CCLE data were analysed [32] (electronic supplementary material, figures S2 and S3*a*,*b*). Overall, BACH1 is likely associated with a more mesenchymal, glycolytic, ferroptosis-sensitive and immune-evasive phenotype, but RKIP tends to promote an epithelial, immune-sensitive and ferroptosis-insensitive cell state dependent on OXPHOS and FAO.

### 2.2. Association of RKIP and BACH1 with EMT and tamoxifen resistance in ER+ breast cancer

**Figure 2.**
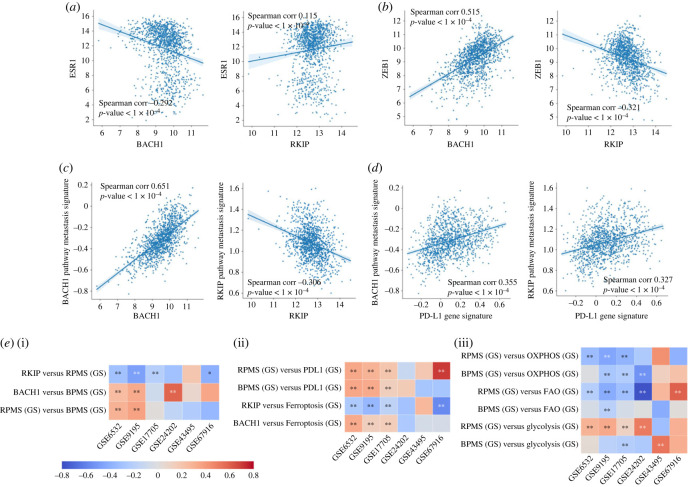
Antagonistic trends of RKIP and BACH1 with EMT and associated axes in breast cancer. (*a*,*b*) Scatter plots showing correlations of RKIP and BACH1 with ESR1, ZEB1 in TCGA breast cancer samples. (*c*,*d*) The same as (*a*,*b*) but for RKIP pathway metastasis signature (RPMS), BACH1 pathway metastasis signature (BPMS). (*e*) Heatmap depicting Spearman's correlation coefficient in 6 ER+ breast cancer datasets: GSE17705, GSE24202, GSE43495, GSE6532, GSE67916, GSE9195. (i) Correlation of RKIP and BACH1 with BPMS, RPMS; (ii) correlation with ferroptosis and PD-L1 gene signatures (GS); (iii) correlation of BPMS and RPMS with metabolic axes OXPHOS, FAO, glycolysis. In these heatmaps, **p-value < 0.05, *p-value < 0.1 (Spearman's correlation analysis).

Next, we focused on breast cancer, given the earlier observations about the mutually antagonistic roles of RKIP and BACH1 in breast cancer [14,33,34]. We first analysed how RKIP and BACH1 correlate with *ESR1*—the gene encoding estrogen receptor alpha (ER*α*). Higher levels of ER*α* are often associated with improved response to anti-estrogen therapies such as tamoxifen and better patient survival [35]. In TCGA breast cancer samples, we observed that ESR1 correlated positively with RKIP (*ρ* = 0.11), but negatively with BACH1 (*ρ* = −0.29) (figure 2*a*). ZEB1, an EMT-inducing transcription factor (EMT-TF), has been shown to hyper-methylate the ER*α* promoter and confer tamoxifen resistance [36]. Thus, we evaluated the association of RKIP and BACH1 with ZEB1. ZEB1 correlated significantly positively with BACH1 (*ρ* = 0.52) and negatively with RKIP (*ρ* = −0.32) (figure 2*b*). Similarly, BACH1 correlated positively with another EMT-TF (SNAI2) and an EMT marker vimentin (VIM) but negatively with OVOL2, an MET-inducing transcription factor [37], showing opposite trends to those seen for RKIP (electronic supplementary material, figure S5*a*). These results indicate that BACH1 associates with EMT, but RKIP associates with MET in breast cancer. To investigate the associations of RKIP and BACH1 with metastasis, we used their respective pathway metastasis signatures [25,38]. In TCGA breast cancer samples (figure 2*c*) and in other TCGA cancer types (electronic supplementary material, figure S5*b*), ssGSEA scores of RKIP pathway metastasis signature (RPMS) correlated negatively with RKIP, while those of BACH1 pathway metastasis signature (BPMS) correlated positively with BACH1. Consistently, RPMS and BPMS ssGSEA scores correlated positively with one another in a pan-cancer manner (electronic supplementary material, figure S5*b*), thus endorsing the pro-metastatic role of BACH1 and an anti-metastatic role of RKIP.

We next focused on six ER+ breast cancer datasets that we had previously analysed from the perspective of EMT and tamoxifen resistance driving each other in ER+ breast cancer [12]: GSE6532, GSE9195, GSE17705, GSE24202, GSE43495 and GSE67916. We noticed resonating trends in these datasets as noted in TCGA samples: RKIP inversely correlated with ssGSEA scores of RPMS and ferroptosis gene signature, while BACH1 correlated positively with ssGSEA scores of BPMS and ferroptosis signature (figure 2*e*(i)(ii)). Further, both RPMS and BPMS scores correlate positively with those of PD-L1 signature but negatively with OXPHOS (figure 2*e*(ii)(iii)), reminiscent of observations of PD-L1 activity being anti-correlated with OXPHOS across carcinomas [39]. Across these datasets, RPMS correlates positively with glycolysis but negatively with FAO, while BPMS shows relatively weaker trends (figure 2*e*(iii)). Similar trends were observed when CCLE data were analysed (electronic supplementary material, figures S3*c*, S4 and S6). This analysis further strengthens the pan-cancer trends that we noticed earlier in terms of the antagonistic association of RKIP and BACH1 with multiple axes of phenotypic plasticity.

### 2.3. Underlying gene regulatory networks reveal RKIP and BACH1 as members of mutually antagonistic ‘teams’

To understand the mechanisms that can explain the observations about RKIP and BACH1 showing opposite trends with respect to metastatic propensity, we identified a minimal core underlying GRN in breast cancer that incorporates the feedback loops that RKIP and BACH1 are involved in with key players of EMT, tamoxifen resistance and stemness, building on our previous efforts to connect these axes of plasticity [12,40]. This network is not meant to be exhaustive in terms of connections RKIP and BACH1 have with players controlling these axes of plasticity but demonstrates a core network structure that may be sufficient to explain the correlation-based observations of RKIP and BACH1 noted earlier.

**Figure 3.**
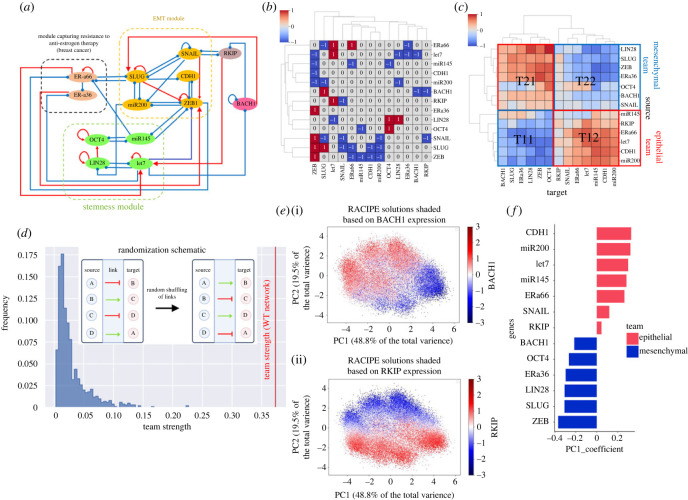
Gene regulatory network underlying antagonistic roles of RKIP and BACH1 as members of opposite ‘teams’. (*a*) Regulatory network showing interconnections of RKIP and BACH1 with the modules of EMT, tamoxifen resistance and stemness. The genes shown here are the nodes of the network while the regulatory links are the edges. Red links indicate activation; blue links indicate inhibition. (*b*) Adjacency matrix for the network shown in (*a*). (*c*) Influence matrix for path length = 8 for network shown in (*a*). (*d*) Network randomization schematic (inset) and histogram of team strength of 1000 random networks generated. The red line indicates the team strength of the wild-type (WT) network, i.e. shown in (*a*). (*e*) Scatterplots of principal component 1 (capturing 48.8% variance) and principal component 2 (capturing 19.5% variance) shaded by BACH1 and RKIP levels. (*f*) Bar plot showing the PC1 loading coefficients of different genes in the network. The bar colours show the same antagonistic team behaviour of the teams identified in (*c*).

Broadly speaking, this network has three core modules: EMT, tamoxifen resistance, and stemness (figure 3*a*). The EMT module comprises EMT-TFs SNAIL, SLUG and ZEB1, EMT-inhibiting micro-RNA-200 family, and E-cadherin (*CDH1*), a key cell–cell adhesion molecule that maintains tight junctions among epithelial cells [12]. The stemness module is composed of OCT4, LIN28, let-7 and miR-145. LIN28 and let-7 engage in a mutually inhibitory loop, and so do miR-145 and OCT4 [40,41]. In the tamoxifen resistance module, we include ER*α*66 and ER*α*36, two variants of estrogen receptor (*ESR1*)—ER*α*66 is associated with tamoxifen-sensitive cell state, while elevated levels of ER*α*36 drive resistance to tamoxifen in breast cancer cells [42]. ER*α*66 can suppress ER*α*36 levels [43] directly, while ER*α*36 can inhibit ER*α*66 by activating ZEB1 [12]. Other links across modules involve the inhibition of LIN28 by miR-200 [44] and inhibition of Er*α*66 by miR-145 [45], as well as mutual inhibition between miR-145 and ZEB1 [41], and that between SLUG and ER*α*66 [12]. RKIP and BACH1 associate with these modules through the following links: (a) BACH1 can self-inhibit and activate SLUG [46], (b) RKIP and SNAIL inhibit each other [47,48], and (c) BACH1 represses RKIP directly, while RKIP inhibits BACH1 via let-7 [14].

Before simulating the emergent dynamics of this network, we generated a corresponding adjacency matrix. This matrix—showing only the direct links (both activation and inhibition) between different nodes in a network—is rather spare (figure 3*b*). Because the influence of one node on another can also be mediated through indirect links, we derived the influence matrix for this network [49] up to a path length of eight edges and performed hierarchical clustering (figure 3*c*). The clustering revealed the presence of two ‘teams’ such that members within a team effectively activated one another, while members across two teams effectively inhibited each other. One team consisted of players corresponding to an epithelial phenotype (let-7, miR-200, CDH1), while the other team comprised drivers of EMT (ZEB, SNAIL, SLUG) and stemness (LIN28, OCT4). As expected, ER*α*66 was found to be a part of the MET-promoting ‘team’ while ER*α*36 belonged to an EMT-driving one. Similarly, RKIP and BACH1 were found in two opposite ‘teams’, consistent with our previous TCGA analysis showing RKIP to be pro-epithelial but BACH1 to be pro-EMT.

Next, we quantified the team strength for this influence matrix and found it to be 0.37 (on a scale of 0 to 1). To examine whether this observation of ‘teams’ is unique to this network, we generated 1000 random networks by shuffling the edges among the nodes (following the randomization schematic; figure 3*d* inset) and determined their corresponding team strength. We observed the team strength of the wild-type (WT) network to be much greater than that of all 1000 randomly generated networks (figure 3*d*). This observation suggests that the organization of these molecular players into ‘teams’ in this network is not a coincidence, but rather a unique topological signature that can possibly facilitate specific couplings between cellular behaviour, such as the association of EMT with stemness and tamoxifen resistance [50,51] and opposite roles of RKIP and BACH1 in EMT [46,52].

Further, we simulated the dynamics of this GRN by representing the regulatory interactions through a set of coupled ordinary differential equations across an ensemble of kinetic parameters and initial conditions using a tool called RACIPE [53]. The output of RACIPE is the set of steady-state solutions obtained, which are then z-normalized for a better comparison across the expression patterns. Principal component analysis (PCA) performed on RACIPE solutions indicated that over 60% of the variance could be captured by the first two principal components (PCs) (electronic supplementary material, figure S7*a*): PC1 accounted for 48.8%, while PC2 accounted for 19.5%. Colouring the PCA plots based on z-normalized levels of RKIP and BACH1 revealed that clusters showing higher BACH1 levels had lower RKIP levels and vice versa (figure 3*e*). Next, we plotted the loading coefficients of each node in the network along the PC1 axis to understand their contributions to PC1 variance. We observed that genes earlier identified to be a part of the epithelial team from the influence matrix analysis had PC1 coefficients greater than zero, while all of them in the mesenchymal team (with SNAIL being the only exception) had these coefficients less than zero. This analysis reveals that PC1 is largely able to recapitulate the members belonging to two different ‘teams’ identified via the influence matrix. The existence and constitution of these ‘teams’ are further validated by a largely bimodal distribution of all network nodes, PCA correlation circle, and pairwise correlation matrices showcasing positive correlation among members within a team and negative across ‘teams’ (electronic supplementary material, figure S7*b*,*c*).

### 2.4. Regulatory network dynamics explains the association of BACH1 with stem-like cell state

**Figure 4.**
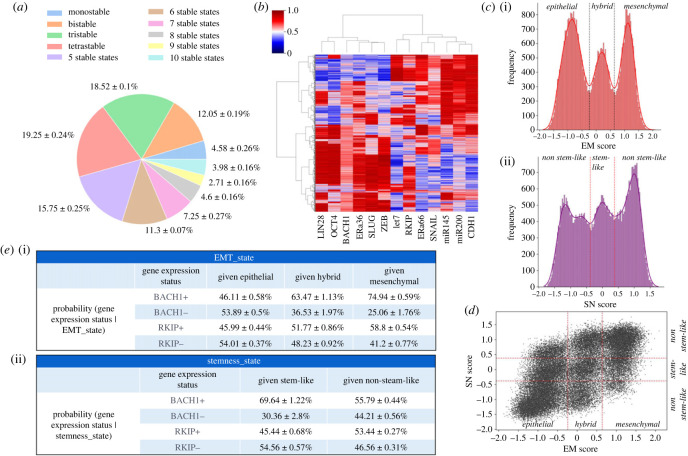
Association of RKIP and BACH1 with EMT and stemness states. (*a*) Pie chart depicting the number of parameter sets giving rise to different number of stable states. Data from 3 independent replicate RACIPE simulations shown as mean ± standard deviation. (*b*) Heatmap of stable steady-state solutions for network shown in figure 3*a*, simulated from RACIPE. Colour bar represents the relative levels of individual genes (z-normalized and log2 expression values). (*c*) (i) Kernel density plot of EM score (= (ZEB + SLUG – miR200 – CDH1)/4). Red dotted lines indicate the segregation of epithelial, hybrid and mesenchymal phenotypes based on minima of the fitted Gaussians. (ii) Kernel density plot of SN score (= (OCT4 +LIN28 – mir145 – let7)/4). Red dotted lines indicate the segregation of stem-like and non-stem-like phenotypes based on minima of the fitted Gaussians. (*d*) Scatterplot of all RACIPE solutions, projected on SN score versus EM score axes. Segregation done based on thresholds identified in (*c*)(i, ii). (*e*) (i) Table depicting the conditional probability percentage of gene expression status given the epithelial, mesenchymal or hybrid phenotypes. (ii) Same as (i) but given stem-like and non-stem-like categorization. BACH1 (or RKIP) +ve indicates z-score expression of BACH1 (or RKIP) > 0, and –ve indicates it < 0.

Next, we investigated whether this regulatory network is capable of multi-stable behaviour, i.e. allowing for the coexistence of multiple cell states that can reversibly switch among themselves. We segregated the parameter sets generated by RACIPE based on their corresponding number of stable states (figure 4*a*). Our analysis revealed that only 4.58% of solutions correspond to a mono-stable state; the remaining 95.42% of solutions are associated with two or more coexisting cell states, highlighting the underlying multi-stable dynamics of this GRN that can support phenotypic plasticity.

Further, we plotted the ensemble of stable states obtained from RACIPE as a heatmap (figure 4*b*). It showed expected clustering patterns and the dominance of two distinct states—(high LIN28, high OCT4, high BACH1, high ER*α*36, high SLUG, high ZEB, low let-7, low ER*α*66, low SNAIL, low miR-145, low miR-200, low CDH1) and (low LIN28, low OCT4, low BACH1, low ER*α*36, low SLUG, low ZEB, high let-7, high ER*α*66, high SNAIL, high miR-145, high miR-200, high CDH1)—thus recapitulating the ‘teams’ seen in influence matrix of the GRN. These two states correspond to a mesenchymal, stem-like, tamoxifen-resistant and epithelial, non-stem-like, tamoxifen-sensitive state, respectively. Interestingly, higher levels of RKIP were seen in most RACIPE solutions that correspond to an epithelial cell state, as well as a subpopulation of mesenchymal cell-state subpopulation. This observation is consistent with a lower magnitude of the loading coefficient of RKIP in explaining PC1 variance as compared to that of BACH1 (figure 3*d*). Thus, our simulations suggest that BACH1 associates strongly with a partial/full EMT state relative to the association of RKIP with an epithelial one.

To better elucidate the functional mapping of phenotypes from the RACIPE expression data, we defined an epithelial mesenchymal (EM) score as EM score = (ZEB1 + SLUG − miR200 − CDH1)/4. ZEB1 and SLUG are characteristic EMT markers, while miR-200 and CDH1 are those for an epithelial state. The higher the EM score, the more mesenchymal the corresponding cell state is. A histogram of EM scores thus obtained across all RACIPE solutions indicated three distinct populations, which can be characterized as epithelial, mesenchymal and hybrid ones (figure 4*c*(i)). Similarly, to quantify plasticity along the stemness axes, we defined a stemness (SN) score as SN score = (OCT4 + LIN28 − miR145 − let7)/4. Extremely high or low levels of OCT4 and LIN28 are associated with non-stem-like states [54–56]; thus we ascribe intermediate levels of SN scores to a stem-like state, while very high or low levels of it to a non-stem-like state, based on the histogram (figure 4*c*(ii)).

The z-normalized RACIPE solutions were then projected on a scatter plot with corresponding SN and EM scores as the axes. While the scatter plot showed a general positive correlation between EM and SN scores (*ρ* = 0.701), we noticed associations of all three phenotypes along the EMT axis with both stem-like and non-stem-like states (figure 4*d*). To better understand how RKIP and BACH1 levels are associated with these axes, we divided the RACIPE steady state solution ensemble into BACH1 (+ve) and BACH1 (−ve) (and similarly for RKIP (+ve) and RKIP (−ve)) cases based on their corresponding z-normalized values. We observed that 63.48% of *in silico* cells (considering each steady-state solution of RACIPE as equivalent to a cell in an experimental population-level setting) showing a hybrid E/M phenotype were BACH1 (+ve) (figure 4*e*(i)). Similarly, a mesenchymal cell was three times more likely to be BACH1 (+ve) as compared to BACH1 (−ve) (74.94% versus 25.06% cases) (figure 4*e*(i)). Consistent with experimental observations of partial and/or full EMT phenotypes with stemness [57–59], we found a stem-like cell to be 2.3 times (69.64% versus 30.36%) more likely to be BACH1 (+ve) as compared to being BACH1 (−ve) (figure 4*e*, ii). On the other hand, the RKIP (+ve) or RKIP (−ve) cells were not enriched in any subpopulation along the EMT or stemness axes. Together, we can infer that BACH1 associates with a hybrid E/M and/or mesenchymal stem-like phenotype.

### 2.5. High BACH1 expression levels are associated with worse patient survival in many cancers

**Figure 5.**
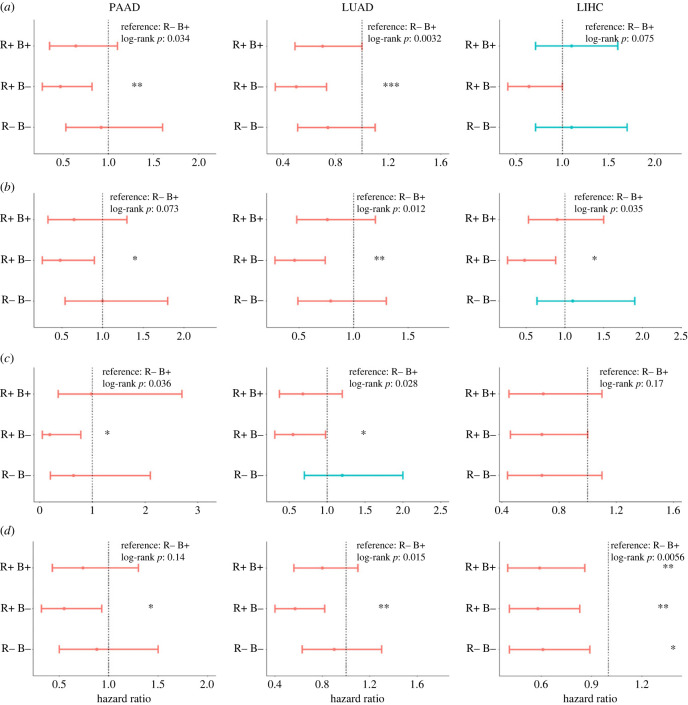
Association of RKIP and BACH1 expression levels with patient survival. Forest plots comparing overall survival (OS) for different combinations of RKIP (low, high) and BACH1 (low, high) when the reference is R− B+ in TCGA samples. *p*-values are based on log-rank test, and those with significant differences *p* < 0.05, *p* < 0.01 and *p* < 0.001 are marked with *, ** and ***, respectively. A global log-rank *p* value has also been reported for the multiple groups. (*a*) Hazard ratios for overall survival in PAAD, LUAD and LIHC, in order. (*b*) Hazard ratios for disease specific survival in PAAD, LUAD and LIHC. (*c*) Hazard ratios for disease-free survival in PAAD, LUAD and LIHC. (*d*) Hazard ratios for progression-free survival in PAAD, LUAD and LIHC.

To identify the effect of RKIP and BACH1 gene expression on the patient survival outcome, we computed the hazard ratios for different combinations of RKIP and BACH1 (R+ B− (RKIP-high, BACH1-low), R+ B+ (RKIP-high, BACH1-high), R− B− (RKIP-low, BACH1-low)) with R− B+ (RKIP-low, BACH1-high) as the reference point. We observed that high BACH1 expression along with low RKIP expression (R− B+) associates with significantly worse overall survival when compared to high RKIP and low BACH1 (R+ B−) phenotype in three different cancers: lung adenocarcinoma (LUAD), pancreatic adenocarcinoma (PAAD) and LIHC (liver hepatocellular carcinoma). Similar trends were recapitulated in the cases of disease-specific survival, disease-free survival and progression-free survival as well. We also noted that the R+ B+ phenotype associates with significantly better progression free survival compared to the RB+ reference phenotype in LIHC. This indicates that higher RKIP expression may be linked to better survival outcomes (figure 5). Pan-cancer observations consistently indicate that low RKIP and high BACH1 expression profile is linked with a worse survival outcome as compared to a high RKIP and low BACH1 one (electronic supplementary material, figure S8).

## 3. Discussion

Cellular decision-making is often governed by mutually inhibitory feedback loops, also called as a ‘toggle switch’. Such network motifs are commonly reported in various decision-making contexts, such as GATA1-PU.1 toggle switch determining the differentiation of a common myeloid progenitor into erythroid cells (GATA1 >> PU.1) or myeloid cells (PU.1 >> GATA1) [60]. Similar antagonism has been witnessed among many EMT and MET driving transcription factors (TFs) such that the MET-TFs (GRHL2, KLF4, OVOL2 etc.) and EMT-TFs (ZEB1, SNAIL, TWIST etc.) mutually repress one another [61–63]. Such mutually antagonistic ‘teams’ of players—also seen in small cell lung cancer GRN [64]—can thus contribute to cellular plasticity [49].

Here, we show that RKIP and BACH1 belong to two antagonistic ‘teams’. While RKIP belongs to the ‘team’ promoting an epithelial and tamoxifen-sensitive cell state in breast cancer, BACH1 is a part of the ‘team’ enabling a mesenchymal and tamoxifen-resistant phenotype. While the antagonistic role of RKIP and BACH1 in EMT has been well reported, their potential involvement in tamoxifen resistance is only beginning to be investigated through mediators such as TANK-binding kinase 1 (TBK1) [65,66]. Consistently, their contrasting roles are emerging in the contexts of ferroptosis [67,68] and metabolic reprogramming [33] in specific cancer types. Particularly, BACH1 can induce ferroptosis and thus contribute to drive lymphatic metastasis in oesophageal squamous cell carcinoma [69]. Similarly, activation of NRF2—a master regulator of antioxidant programmes in cells—stabilizes BACH1 [70]. NRF2 activity can be enhanced by loss of RKIP [71], enabling another ‘toggle switch’ between RKIP and BACH1. NRF2 has also been reported to maintain cells in a hybrid epithelial/mesenchymal (E/M) phenotype [72] and enhance stemness and chemoresistance [71,73]. Thus, our results about association of BACH1 with a hybrid E/M stem-like state unify the previous observations about role of BACH1 in controlling multiple axes of plasticity.

**Figure 6.**
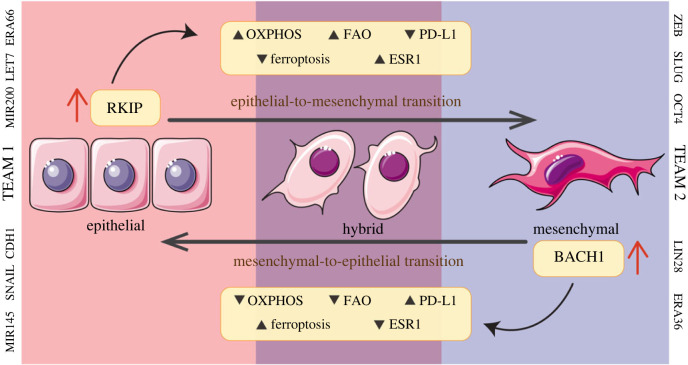
RKIP and BACH1 belonging to separate ‘teams’ driving two opposite phenotypes. Schematic showing the association of RKIP with an epithelial phenotype which has reduced ferroptosis and PD-L1 associated activity, but enrichment of FAO, OXPHOS activity. BACH1 associates with a mesenchymal phenotype that has reduced FAO, OXPHOS activity but higher PD-L1 and ferroptosis activity. In ER+ breast cancer, ESR1 expression associates positively with epithelial phenotype. Members of two antagonistic ‘teams’ to which RKIP and BACH1 belong are also mentioned alongside.

The contrasting roles of RKIP and BACH1 in mediating stemness/dedifferentiation lends further credence to our model simulations. BACH1 can activate CD44, and MAPK signalling in lung cancer stem cells (CSCs) and stimulate lung cancer metastasis; its loss represses metastasis in xenograft models [24,74]. Similarly, BACH1 represses mesendodermal differentiation in embryonic stem cells, maintaining stem-cell identity [75]. Conversely, RKIP can repress NANOG in primary melanocytes, maintaining their differentiation state [76]. Chemical induction of RKIP can degrade SOX2, inhibit tumour growth and promote differentiation of schwannoma into mature Schwann cells [77]. These observations, together with the contrasting roles of RKIP and BACH1 in tumour cell migration [78,79], can explain the association of higher BACH1 levels with enhanced metastasis and poor patient prognosis [21,22,24]. Our results demonstrate that these trends of association of RKIP and BACH1 with clinical outcomes are largely consistent across cancer types as well as survival metrics (overall survival, progression-free survival, disease-free survival), highlighting their behaviour as members of two antagonistic ‘teams’ (figure 6) playing a tug-of-war—a pro-metastatic one (EMT, stem-like, drug-resistant) and an anti-metastatic one.

While ‘teams’ comprising EMT-TFs and MET-TFs have been well established, it remains to be seen whether the molecular factors stabilizing hybrid E/M phenotypes such as NRF2 and *Δ*NP63 form a separate ‘team’ and how antagonistic they are with both EMT-TFs and MET-TFs ‘teams’ [80,81]. Moreover, although the role of RKIP and BACH1 in altering one of the axes of cancer cell plasticity at a time—EMT, stemness, ferroptosis, metabolic switching—has been well studied, their ability to drive adaptation along multiple axes simultaneously due to the impact of ‘teams’ remains to be investigated experimentally, to further lend credence to our model predictions. Finally, given that members of the same ‘team’ can partly compensate for each other's functional contribution, we expect that identifying such modules can help recognize combinatorial inhibitions for a synergistic impact, for instance, as noted for PDGFR and SGK1 inhibition [82].

Overall, our pan-cancer systems-level analysis reveals that RKIP and BACH1 can control multiple axes of plasticity (EMT, metabolic reprogramming, stemness) together in opposite directions, thus explaining their association with patient survival seen across cancer types. Our mechanistic model highlights that such ‘teams’ of players can be an important network motif in coordinating changes along more than one axes of plasticity together, such as EMT, stemness, and metabolic switching. Our results suggest breaking such ‘teams’ as a possible therapeutic avenue to reduce the fitness of metastasizing cells by limiting their phenotypic plasticity trajectories.

## 4. Material and methods

### 4.1. Transcriptomic datasets

We downloaded microarray data from NCBI GEO (GSE17705, GSE24202, GSE43495, GSE6532, GSE67916, GSE9195) using the GEOquery package on R, and mapped the probes to respective genes using their corresponding annotation files. We normalized gene-wise expression matrices on log2 base before further analysis.

We downloaded TCGA gene expression data of 35 different cancer types from the UCSCXena browser, and CCLE gene expression data of 14 different cancer types from https://sites.broadinstitute.org/ccle. Pre-processed datasets were available in transcripts per million (TPM) format and were directly used for analysis. Survival data were analysed using data from TCGA. Relevant cancer samples were split into two groups based on median: high *PEBP1* (gene name for RKIP) versus low PEBP1, and high *BACH1* versus low BACH1. Kaplan–Meier curves for overall survival were plotted using the plotter on the ‘KMPlotter’ website [83]. Additionally, ‘coxph’ function in R package ‘survival’ was employed to determine the hazard ratio (HR) and confidence interval (95% CI) for TCGA cohorts, and heatmaps were made using ‘ggplot2’. Normalized single sample gene set enrichment analysis (ssGSEA) scores based on specific input gene signatures (electronic supplementary material, table S1) were calculated for each sample using the ‘GSEApy’ Python package [84]. For correlation analysis between any two variables, Spearman's correlation coefficient has been used, using the ‘spearmanr’ function from the ‘SciPy’ Python library.

### 4.2. EMT scoring metric

KS score is a metric to quantify the extent of EMT based on expression levels of specific epithelial and mesenchymal markers [26]. It uses two gene signatures: KS-Epi (genes associated with an epithelial phenotype) and KS-Mes (genes associated with a mesenchymal phenotype). It plots two cumulative distribution functions (CDFs) based on expression levels of genes in KS-Epi and KS-Mes signatures. The distance between the 2 CDFs is calculated for each value, and the maximum value is taken as the statistic. KS score values lie in the range [−1, 1].

### 4.3. Identification of ‘teams’ of players

An adjacency matrix (Adj) represents the topology of a GRN in the form of a matrix. Rows represent the source node for a particular link, while the columns represent the target nodes. An inhibitory link is represented (in blue) with value −1; an activation link is represented (in red) with value 1. Path length is defined as the number of consecutive links in a path that connects a source node to its corresponding target. In a GRN, the nodes not only influence their direct target (path length = 1) but also other nodes indirectly (path length > 1). To take into consideration these interactions, we define an influence matrix that captures both the direct and indirect interactions between nodes in the network up to a defined path length [49]:

$$\mathrm{Infl}\;=\;\frac{\sum_{l=1}^{l_{\max}}({\mathrm{Adj}}^{l}/{\mathrm{Adj}}_{\max}^{l})}{l_{\max}},$$

where Adj*^l^* represents the adjacency matrix (Adj) multiplied with itself *l* times. Adj_max_ represents the Adj matrix with all inhibition links replaced by activation links. ${\mathrm{Adj}}^{l}/{\mathrm{Adj}}_{\max}^{l}$ represents the element-wise division of values in Adj*^l^* by values in ${\mathrm{Adj}}_{\max}^{l}$.

A positive value indicates activation and a negative influence indicates inhibition. The higher the value in the influence matrix, the higher the influence of that specific source gene on the target. Hierarchical clustering is performed on the influence matrix to identify the clusters of genes functioning similarly. The team strength of each cluster *T*_KL_ is given as

$$T_{\mathrm{KL}}=\frac{\sum_{i\in T_{K},j\in T_{L}}{\mathrm{Infl}}_{ij}}{n_{\mathrm{KL}}}\;,\;\;\;K,L\;\in \;\{1,2\}.$$

Team strength of the entire network *T*_S_ is given as

$$T_{S}=\frac{\sum_{K,L\in \{1,2\}}|T_{KL}|}{4}.\;$$

### 4.4. RACIPE analysis

Random Circuit Perturbation (RACIPE) is a tool used to simulate the dynamics of GRNs. It takes as input the topology of a GRN and generates an ensemble of kinetic models for the given GRN. Here, we simulated the network shown in figure 3*a*—the input file to RACIPE is given as electronic supplementary material, table S2.

For each kinetic model generated for the input topology file, RACIPE samples many initial parameters from the designated range for each parameter. The expression levels of a node in a GRN is determined by a set of ordinary differential equations given below:

$$\frac{dX_{i}}{dt}=g_{X_{i}}\underset{j}{\prod }H^{S}(X_{j},X_{\;ji}^{0},n_{\;ji},\lambda_{\;ji})-k_{X_{i}}X_{i}.\;$$

Here, $X_{i}\;$ is concentration of gene product of the gene node $X_{i}\;$ part of GRN and $dX_{i}/dt$ is the rate of change of gene expression with respect to time. $G_{X_{i}}$ is basal production rate; $k_{X_{i}}$ is basal degradation rate of gene product $X_{i}$. $H^{S}$ represents shifted Hill function that models the activation and inhibition links towards this $\left(X_{i}\right)$ gene node in the GRN. $n_{\;ji}$ is Hill function coefficient and $X_{\;ji}^{0}$ is threshold value of the Hill equation. $X_{j}$ is concentration of gene product $X_{j}$, where $X_{j}$ is a node either activating or inhibiting $X_{i}$. Since our GRN had 13 nodes, *i* and *j* can take integer values between 1 and 13. $\lambda_{\;ji}$ is fold change parameter. Throughout this study, we simulated GRNs for 10 000 parameter sets. RACIPE gives log_2_ normalized steady-state gene expressions of each gene product as output. This output steady state gene expression data was then z-normalized and used for analysis. To check for bimodality in solutions obtained from RACIPE, we used Sarle's bimodality coefficient:

$$\mathrm{BC}\;=\;\frac{s^{2}\;+\;1}{k\;+\;3\cdot ({\left(n-1\right)}^{2}/((n-2)(n-3)))}.$$

Here, *s* is the skew of the distribution and *k* is the kurtosis. BC can take values between 0 and 1, with BC values greater than 0.55 indicating bimodal distribution of gene expression [85]. SciPy library has been used to calculate kurtosis (*k*) and skew (*s*) of the distribution.

### 4.5. Survival analysis

Survival data were obtained from TCGA cohort of patients for all available cancer types. The samples were categorized into RKIP high (R+) and RKIP low (R−) as well as BACH1 high (B+) and BACH1 low (B−) groups based on median of the respective scores of the samples. The RKIP (R+/R−) and BACH1(B+/B−) states were combined to study their synergistic effect on survival. With R− B+ serving as the reference, we calculated the hazard ratios for various combinations of RKIP and BACH1 (R + B−, R + B+, R− B−) by performing Kaplan–Meier analysis using R package ‘survival’. A log-rank test was used to compute the *p*-values between the reference and other groups. The reported hazard ratio (HR) and confidence interval (95% CI) were determined using Cox regression using the ‘coxph’ function. Statistical analysis, survival analysis, and plots were all performed in R version 4.3.0. Forest plots were plotted using the ‘ggforest’ function from the ‘survminer’ package.

## Data Availability

GSE datasets analysed were obtained directly from GEO (Gene Expression Omnibus). The codes used are available from the GitHub repository: https://github.com/saishyam1/RKIP_BACH1_Data_Codes. Supplementary material is available online [86].
